# TumorGAN: A Multi-Modal Data Augmentation Framework for Brain Tumor Segmentation

**DOI:** 10.3390/s20154203

**Published:** 2020-07-28

**Authors:** Qingyun Li, Zhibin Yu, Yubo Wang, Haiyong Zheng

**Affiliations:** 1 College of Information Science and Engineering, Ocean University of China, Qingdao 266100, China; lliqingyun11@gmail.com (Q.L.); yuzhibin@ouc.edu.cn (Z.Y.); zhenghaiyong@ouc.edu.cn (H.Z.); 2 School of Life Science and Technology, Xidian University, Xi’an 710071, China

**Keywords:** medical image augmentation, generative adversarial network, brain tumor segmentation, image-to-image

## Abstract

The high human labor demand involved in collecting paired medical imaging data severely impedes the application of deep learning methods to medical image processing tasks such as tumor segmentation. The situation is further worsened when collecting multi-modal image pairs. However, this issue can be resolved through the help of generative adversarial networks, which can be used to generate realistic images. In this work, we propose a novel framework, named TumorGAN, to generate image segmentation pairs based on unpaired adversarial training. To improve the quality of the generated images, we introduce a regional perceptual loss to enhance the performance of the discriminator. We also develop a regional $L_{1}$ loss to constrain the color of the imaged brain tissue. Finally, we verify the performance of TumorGAN on a public brain tumor data set, BraTS 2017. The experimental results demonstrate that the synthetic data pairs generated by our proposed method can practically improve tumor segmentation performance when applied to segmentation network training.

## 1. Introduction

An accurate tumor segmentation model is pivotal for early tumor determination and radiotherapy arrangement [1]. Traditionally, tumor segmentation is performed by finding a mapping function between a real medical image (e.g., an MRI image) and a semantic label image of a real tumor, as depicted by medical professionals. With the rapid development of medical imaging equipment, substantial effort has been directed towards the research of segmentation tasks using multi-modal data pairs [2,3,4,5]. Generally, multi-modal data can lead to a better performance result, as compared to approaches based on a single modality, because more information about the tumor could be captured by different imaging methods [6]. Motivated by the success of deep learning, researchers soon applied deep neural networks to solve various medical imaging-related problems [7,8,9]. However, unlike classification, labeling medical images for segmentation is challenging, as it is time-consuming and requires medical specialists [10]. Labeling multi-modal medical images further increases the complexity of such a task. The lack of properly labeled tumor masks limits the potential of data-driven medical image segmentation such as those involving deep learning-based methods. Data augmentation (e.g., rotation, flipping) is one possible way to expand a data set with limited labeled samples. However, these methods are insufficient to represent the variations of shape, location, and pathology.

Recently, many researchers have used generative adversarial networks (GANs) for image synthesis and data augmentation. Although the earlier variants of GANs can only generate images from random noise [11], conditional GAN-based image-to-image translation models provide new solutions for pixel-wise image augmentation [12]. Many powerful GAN-based image-to-image variants have been proposed [13,14], which can generate realistic images by considering an input image and a given condition. In fact, some popular image-to-image translation frameworks such as Pix2pix [15] or CycleGAN [16] have already shown potential for pixel-wise image augmentation by converting an image only including semantic information to a realistic image. However, we still need to address two challenges before such methods can be applied to multi-modal medical image augmentation. The first challenge is a lack of source data, which means that we need to generate reasonable semantic labels first before feeding them into the image-to-image translation models. An incorrect lesion area may lead to an useless augmentation output. Furthermore, we need to guarantee the quality of the synthesized images without enough ground truth during the augmentation stage due to the absence of image pairs. Hence, to obtain a realistic medical image at the pixel level, adversarial training is necessary but requires further improvement.

**Figure 1:**
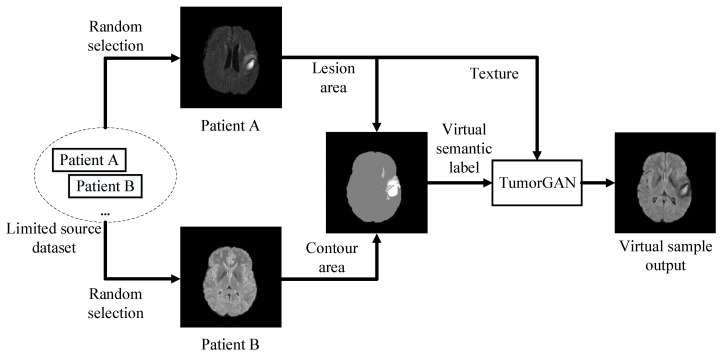
An overview of TumorGAN-based medical image augmentation.

To generate realistic paired data with limited source data for medical image augmentation, we synthesize a pixel-wise semantic label image by combining the lesion area with the brain contour from two real medical images. Then, we feed the virtual semantic label image with texture image from patient A to generate the corresponding output, as displayed in Figure 1. By doing so, in an ideal case, we can obtain $n^{2}-n$ virtual samples from *n* patients for data augmentation. Furthermore, the validity of the synthetic semantic label images can be guaranteed, as both the contour and lesion area come from real samples. The synthetic semantic label image can also provide an attention region to help us build a regional perceptual loss, as well as a regional $L_{1}$ loss, in order to train the image-to-image translation model with a regional ground truth and improve the generalization performance. To further enhance the efficiency of adversarial learning, we include an additional local discriminator co-operating with the main discriminator.

In this work, we have the following contributions:

- We propose an image-to-image translation framework called TumorGAN, which can synthesize $n^{2}-n$ virtual image pairs from *n* real data pairs for brain tumor segmentation. Our experimental results show that TumorGAN is able to augment brain tumor data sets and can improve the performance of tumor segmentation for both single-modality data and multi-modal data.
- We design a region perceptual loss and an $L_{1}$ loss based on attention areas provided by the semantic labels to preserve the image details.
- We included an extra local discriminator co-operating with the main discriminator, in order to increase the efficiency of the discriminator and help TumorGAN to generate medical image pairs with more realistic details.

## 2. Related Work

### 2.1. Brain Tumor Segmentation

Brain tumor segmentation is a challenging task, due to the structural complexity of brain tissue [17]. Many brain tumor segmentation approaches have been proposed. Researchers have earlier developed brain segmentation approaches based on contours [18], regions [19], statistics [20], and some traditional machine learning models [21]. As deep learning approaches have become more popular, more and more deep learning-based brain tumor segmentation methods have also been presented [22,23]. As an increasing number of multi-modal data sets, like BraTS challenge [24], have been released, several deep learning based multi-modal medical image segmentation methods [25,26] have also been proposed. As image-to-image translation frameworks have become popular for segmentation tasks, GAN-based image-to-image translation provides another solution for brain tumor segmentation. In this paper, we take advantage of a GAN-based image-to-image framework for multi-modal brain tumor processing. Differing from previous approaches, we aimed at applying the framework to the pre-processing stage for multi-modal brain tumor data augmentation. Our main aim is to use the augmented data to improve the robustness and performance of the segmentation model.

### 2.2. Generative Adversarial Network Based Medical Image Augmentation

Collecting and labeling medical images is a difficult task, due to the lack of experts and privacy concerns, thus limiting the application of supervised deep learning approaches [27]. Typically insufficient numbers of medical images requires the development of medical image augmentation methods. Earlier simple geometric deformation methods, including scaling, rotation, and flipping, have been employed to increase the variety of data sets [28]; however, such augmentation methods cannot represent the variations in the shape, location, and pathology of a brain tumor.

The success of generative adversarial networks (GANs) has provided a general solution for image augmentation [29]. A straightforward way of applying a GAN to medical image argumentation is to use the noise-to-image structure, which originates from the vanilla GAN and some variants [11,14,30], generating images from one-dimensional vectors [31]. However, these approaches cannot obtain a pixel-wise matching between two images. Another way is to take the advantage of the image-to-image translation framework for medical image augmentation. Earlier image-to-image translation models such as Pix2pix [15] need paired training data, which are expensive to obtain for medical images. CycleGAN, which can translate images from one domain to another with unpaired data [16], is more popular in solving medical image augmentation problems. Unpaired image-to-image translation models can even translate medical imaging across different modalities, such as MRI to CT translation [32,33,34]. Image-to-image frameworks have also been widely used for medical image quality enhancement or denoising [35,36]. Unlike the methods for medical image processing mentioned above, which attempt to build a mapping function between two modalities, our proposed method, TumorGAN, aims to build a mapping function between an edited semantic label image and a modality (flair, t1, t1ce, and t2).

## 3. Method

**Figure 2:**
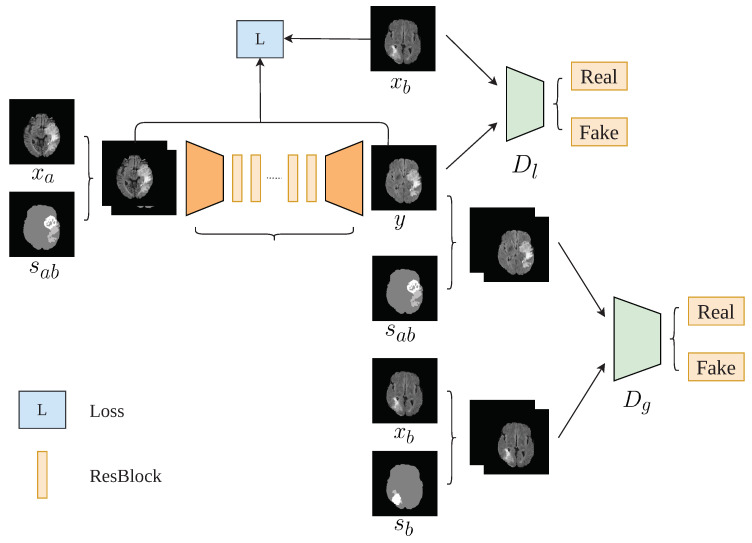
TumorGAN architecture. We use one generator *G* and two discriminators $D_{g}$ and $D_{l}$. The output of *G* is entered into the different discriminators in different ways to determine whether it is real or fake; $s_{ab}$ is a semantic segmentation label corresponding to *y* and $s_{b}$ is a semantic label corresponding to $x_{b}$.

In the following, we introduce a brain data augmentation approach called tumorGAN, followed by a graphical description shown in Figure 2.

### 3.1. Synthesis of Semantic Label Image

**Figure 3:**
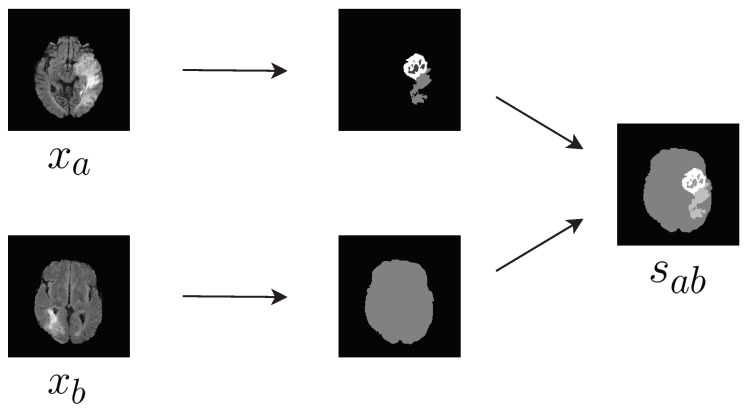
Composition of the label $s_{ab}$.

The contour and shape of brain tumor areas are complicated, due to the underlying pathology and anatomy. Thus, it would be difficult to synthesize a reasonable tumor image directly. Instead, we developed a way to design the tumor image from existing tumor shapes. Let *x* denote a brain image from a given imaging modality (i.e., FLAIR, T1, T2ce, and T2), and $x_{a}$ and $s_{a}$ denote a brain image and the corresponding semantic label for patient *A*, respectively. We can create a new virtual semantic label image $s_{ab}$ by combining the tumor area from $s_{a}$ and the brain background from $s_{b}$ (Figure 3). In other words, our virtual semantic label image tries to mimic a case in which patient *B* has the same lesion as patient *A*.

### 3.2. Architecture

**Figure 4:**
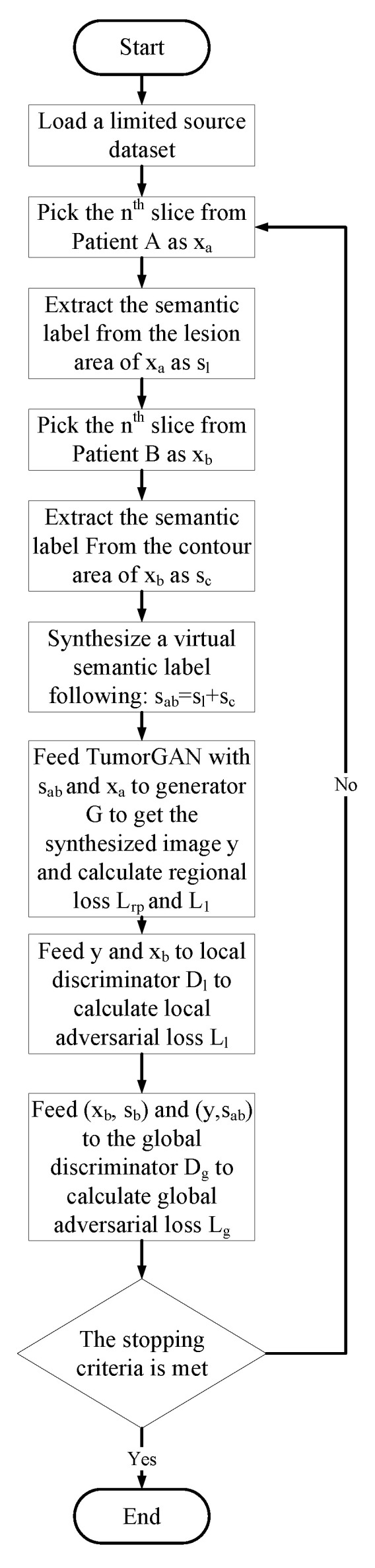
Flow chart of the proposed method.

Similar to most GAN implementations, TumorGAN includes two parts: a generator *G* and a pair of discriminators, $D_{g}$ and $D_{l}$. We follow our previous work [37] to build the generator *G* and inherit the discriminator structure from Pix2pix [15] to form the global discriminator $D_{g}$. As shown in Figure 2, the generator *G* is fed a segmentation label image $s_{ab}$ concatenated with a brain image $x_{a}$ from a given modality (i.e., flair, t1, t1ce, or t2) to generate a synthetic image *y*. The segmentation label provide the outline information include brain background and tumor, and the brain image provide concrete tumor information.We can obtain a brain image $x_{a}$ from a slice of a patient A, and we synthesize a segmentation label image following the method described in Figure 3. Another brain image $x_{b}$ from patient B is used for adversarial and local perceptual loss calculation. To make the *y* fit the semantic label image $s_{ab}$, we designed a regional $L_{1}$ loss, as well as regional perceptual loss $L_{rp}$. Inspired by the local–global discriminator framework [38], we included an additional discriminator, $D_{l}$, to improve the generation performance in terms of fine details. The input size of $D_{l}$ is a 64×64 image cropped from *y* or $x_{b}$. The flow chart of our method is shown in Figure 4.

### 3.3. Formulation

**Figure 5:**
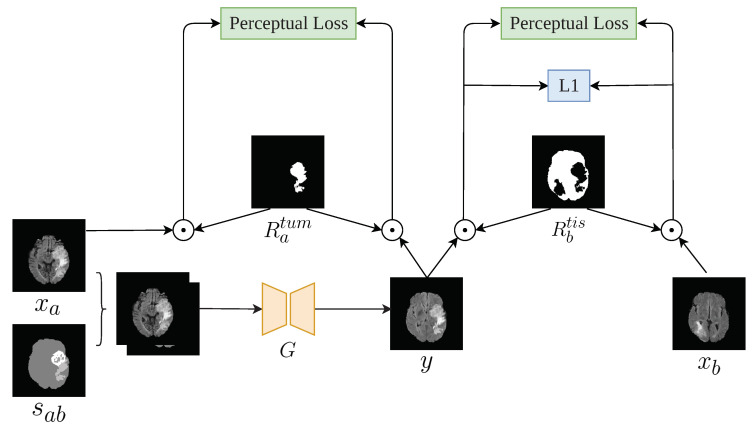
Computation details of the perceptual loss and $L_{1}$ loss. $R_{a}^{tum}$ is a mask for the *x* tumor; $R_{b}^{tis}$ is a mask of the $x_{b}$ tissue, except for the $x_{b}$ and $x_{a}$ tumor sites.

To generate a realistic brain image for each modality, we need to handle the generation task of brain tissue area and the tumor area simultaneously. To achieve this goal, we developed a regional perceptual loss from the perceptual loss [39], as follows:

(1) $$\begin{array}{c} L_{rp}=\lambda_{1}E{\left[\phi_{3,4}\left(y^{tis}\right)-\phi_{3,4}\left(x_{b}^{tis}\right)\right]}^{2}+\\ \lambda_{2}E{\left[\phi_{4,4}\left(y^{tis}\right)-\phi_{4,4}\left(x_{b}^{tis}\right)\right]}^{2}+\\ \lambda_{3}E{\left[\phi_{4,4}\left(y^{tum}\right)-\phi_{4,4}\left(x^{tum}\right)\right]}^{2}, \end{array}$$

(2) $$y^{tis}=y\cdot R_{b}^{tis},$$

(3) $$y^{tum}=y\cdot R_{a}^{tum},$$

where $y^{tis}$ and $y^{tum}$ are the tissue region $R_{b}^{tis}$ and the tumor region $R_{a}^{tum}$ of the output image *y*, respectively; and $\phi_{3,4}$ are the feature maps obtained by the fourth convolutional layer before the third max-pooling layer in VGG-19 [40]. The parameters of λ are set as $\lambda_{1}:\lambda_{2}:\lambda_{3}=1:100:100$. The implementation details of the point multiplication operation can be found in Figure 5.

As the healthy tissue area usually occupies a large area, as compared to the tumor lesion, we adopt a regional $L_{1}$ loss to improve the tissue texture:

(4) $$L_{1}=E[∥y^{tis}-x_{b}^{tis}{∥}_{1}].$$

**Figure 6:**
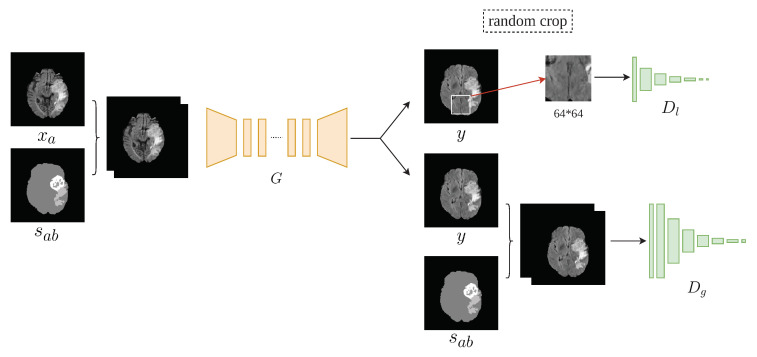
Details of the generator and discriminator structures. $D_{g}$ is the global discriminator and $D_{l}$ is the local discriminator. The input of $D_{l}$ is a randomly cropped section of *y*.

Finally, an extra local discriminator was introduced, which co-operates with the vanilla discriminator (Figure 6) and encourages the generator to generate more realistic images. We designed our local–global adversarial loss function based on the least squares adversarial loss [41]:

(5) $$L_{adv}=L_{g}+L_{l},$$

(6) $$\begin{array}{c} L_{g}\left(D_{g},G\right)=E{\left[\left(D_{g}\left(x_{b},s_{b}\right)\right)-1\right)}^{2}]\\ +E[{\left(D_{g}\left(y,s_{ab}\right)\right)}^{2}], \end{array}$$

(7) $$L_{l}\left(D_{l},G\right)=E{\left[\left(D_{l}\left(x_{b}^{local}\right)\right)-1\right)}^{2}]+E\left[{\left(D_{l}\left(y^{local}\right)\right)}^{2}\right],$$

where $L_{g}$ and $L_{l}$ are the adversarial losses of the global discriminator and the local discriminator, respectively; $x_{b},s_{b}$ is the concatenation of the $x_{b}$ and the corresponded semantic label $s_{b}$; and $y^{local}$ is the randomly cropped region of *y*.

The total loss function can be given as:

(8) $$L=\lambda L_{rp}+\mu L_{1}+\gamma L_{adv},$$

where λ, μ, and γ denote the objective weights; in our experiment, we used a ratio of 1:1000:1000 for these.

## 4. Experiment

To prove the efficiency of the proposed TumorGAN, we first applied TumorGAN on the BraTS 2017 data set to increase the sample number. Then, we validated the usefulness of the synthetic data by using them as part of the training data for several segmentation models, including the cascade model [42], U-Net [43] and deeplabv3 [44].

### 4.1. Implementation Details

The generator structure was derived from CycleGAN, which consists of nine residual blocks in the bottleneck. The local–global discriminators are similar, but with one less convolutional layer due to the reduced size of the input image. The detail of the structure are as follows:

**Generator:**CIR64F7−CIR128F3−CIR256F3−Res256−Res256−Res256−Res256−Res256−Res256−Res256−Res256−Res256−DCIR128F3−DCIR64F3−C1F7;

**Global discriminator:**CLR64F4−CILR128F4−CILR256F4−CILR512F4−CILR512F4−CILR512F4−C1F4; and

**Local discriminator:**CLR64F4−CILR128F4−CILR256F4−CILR512F4−CILR512F4−C1F4,

where CIRmFn in the generator means the Convolutional–InstanceNorm–ReLU layer with *m* n×n filters, Res256 means a residual block with 256 3×3 filters, and the last layer of the generator uses Sigmoid as the activation function. CILRjFk in the discriminator means the Convolutional–InstanceNorm–LeakyReLU layer with *j* filters of k×k.

### 4.2. Data Set Pre-Processing and Data Augmentation

The Multi-modal Brain Tumor Segmentation (BraTS) Challenge provides an annotated 3D MRI data set. In this work, the experiment was conducted on the BraTs 2017 data set, consisting of four MRI imaging modalities with different characteristics, including FLAIR, T1, T1CE, and T2. The BraTS 2017 data set provides 285 labeled patients, from which we used 226 patients (HGG(high-grade gliomas): 166, LGG(low-grade gliomas): 60) as a training set to train TumorGAN, as well as the segmentation network. The remaining cases (HGG: 44, LGG: 15) were used as the testing set, in order to evaluate the algorithm’s performance. We normalized all input images according to the following equation:

(9) $$I_{N}=\left(I-I_{min}\right)/\left(I_{max}-I_{min}\right),$$

where $I_{min}$ and $I_{max}$ are the minimum and maximum pixel values of the input image, respectively.

The image size for a patient was 240 × 240 × 155. As the tumors were always located in the brain tissue, we took slices from 30 to 110 from each patient and resized them to 256 × 256. We used the pre-trained TumorGAN to generate synthetic brain images as well as semantic labels. The augmentation details are shown in Table 1. Theoretically, we can generate 226×225=50,850 virtual samples with 226 real samples in the training data set. Considering the computing time issue, we generated 226 virtual samples—the same amount of samples—for the training data set, in order to support the semantic segmentation task.

**Figure 7:**
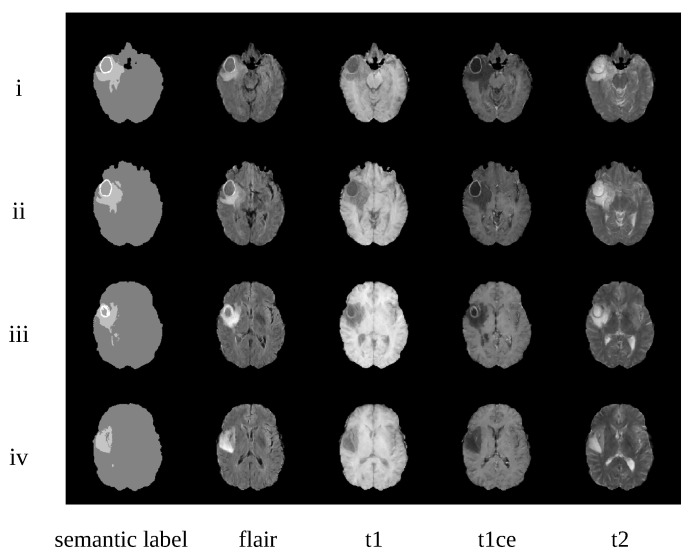
Examples of synthetic images from one patient. i–iv represent slices 52, 58, 66, and 73 from this patient, respectively. Images from the left to the right are the corresponding semantic labels and four synthetic modality images (i.e., flair, t1, t1ce, and t2).

Some synthetic examples are shown in Figure 7. We can observe that the synthesized images are well-matched to the semantic labels. Furthermore, different modality brain images have different features. For example, the T1-CE (t1-weighted contrast-enhanced) modality can ensure that the tumor core is brighter, in order to distinguish the tumor core and edema regions. This can also been seen in our augmentation data.

### 4.3. Qualitative Evaluation

**Ablation Study**

**Figure 8:**
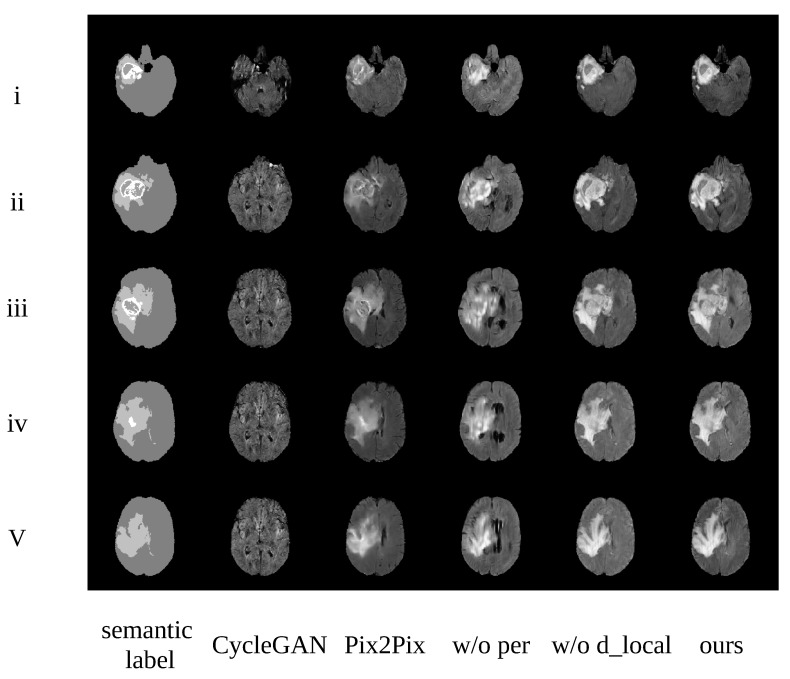
Examples generated from different methods. i–v represent slices 50, 60, 70, 80, and 90 from this patient, respectively. Images from the left to the right are the corresponding semantic label, CycleGAN, Pix2Pix, TumorGAN without regional perceptual loss, TumorGAN without the local discriminator, and TumorGAN.

We designed an ablation study based on the flair modality, in order to demonstrate the efficiency of each component in our proposed TumorGAN, as shown in Figure 8. The i–v lines denoted the samples from slices 50, 60, 70, 80, and 90, respectively. The first column shows the semantic label; the last column includes the results obtained from TumorGAN; and the forth and the fifth columns contain synthetic images from TumorGAN without regional perceptual loss and local discriminator, respectively. It can be observed that, when we removed the regional perceptual loss, the synthetic image was lacking in detail and became blurred. Many artifacts appeared when we removed the local discriminator (see, e.g., Figure 8 w/o d_local).

**Figure 9:**
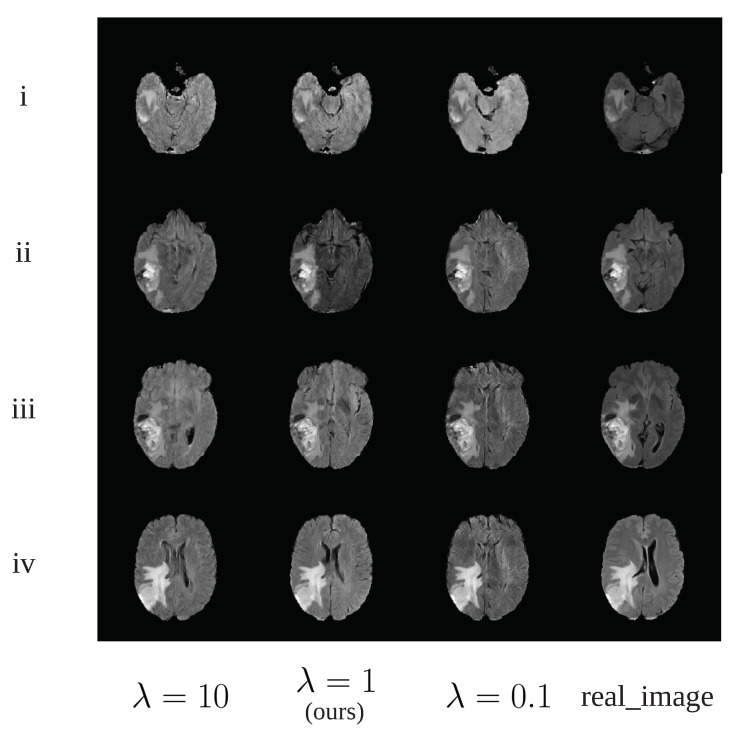
The synthetic image examples using different λ value.

We used a traditional grid search method [45] to obtain the optimal values for λ, μ and γ in Equation (8). Take λ as an example, we searched a possible set {0.001,0.01,0.1,1,10,100,1000} for λ and finally choose λ=1 for the rest of this study. Although we found that all choices of λ produce similar images, it was noticed λ=1 provide better contrast in the generated tumor as shown in Figure 9ii. The procedure for selecting μ and γ is similar to that of λ. Since we treat λ, μ and γ as hyper parameters, identifying the optimal value of these hyper parameters requires re-train the network on the same training dataset many times. Hence, the computational burden is high and we cannot explore a large candidate set for these hyper parameters. It would be possible to obtain a better value for these parameters given enough computational resources.

**Comparison with Baseline**

TumorGAN obtained the best qualitative results, when compared to the baseline CycleGAN (see Figure 8). We used the same data (slices 30–110) as for TumorGAN in the 226 patients as the training data set to train CycleGAN. We created a semantic label image by combining the tumor area and the brain background from the same brain image; in this way, we could acquire the paired data to train the Pix2pix model. We also used the same data in CycleGAN.

To measure the quality of the generated images, we used the Fréchet inception distance (FID) [46] to evaluate the similarities between the real images and the generated samples. TumorGAN obtained FIDs that were favorable, as compared to the baselines (see Table 2). This shows that the proposed method can generate images that closely match the real data distribution. We can see that, compared with the score of CycleGAN (baseline), the FID score of our method was reduced by 50%.

### 4.4. Tumor Segmentation Using Synthetic Data

**Cascaded Net** Wang et al. proposed a cascaded network and acquired the top rank in the BraTS 2018 challenge [42]. Cascaded Net segments the tumor following three stages: In the first stage, the Cascade Net locates and picks out the whole brain tumor area. In the second stage, it removes the useless surrounding tissue area and crops a square tumor region as the input to the next network, in order to segment the tumor core. In the third stage, the third network divides the tumor core into an enhanced region and a non-enhanced region. Finally, multi-view fusion is employed to combine the results from each stage. In our experiment, we only used the axial data for data synthesis. Due to the limited GPU memory, we changed the batch size to 3.

**U-Net** U-Net is a popular deep neural network for medical image segmentation [43]. U-Net has an encoder–decoder structure with several skip connections. Following the structure mentioned in [43], we used four times down-sampling and four times up-sampling to build the U-net in our experiment.

**DeeplabV3** Deeplab and its variants have achieved a great of success in many common semantic segmentation tasks [47,48]. In this work, we used DeeplabV3 as another benchmark for the tumor image segmentation task. DeeplabV3 includes multiple dilated convolutional layers, which expand the field-of-view, and apply a pre-trained network. Furthermore, it augments the Atrous Spatial Pyramid Pooling module proposed previously. In this work, we used resnet50 as the backbone network to implement DeeplabV3.

All the three segmentation models were used for evaluation. The data split for each model is given in Table 1:

The dice score [49] was employed to evaluate the tumor segmentation performance on the testing set. The score is defined as follows:

(10) $$Dice\left(p_{\mathrm{true}},p_{\mathrm{pred}}\right)=\frac{2\cdot \sum p_{\mathrm{true}}\cdot p_{\mathrm{pred}}}{\sum p_{\mathrm{true}}+\sum p_{\mathrm{pred}}+\epsilon },$$

where $p_{\mathrm{pred}}$ is the output and $p_{\mathrm{true}}$ is the segmentation ground truth. The summation is voxel-wise and ϵ is a very small constant to prevent divsion by zero.

#### 4.4.1. Training on Multi-Modal Dataset

The performance of the segmentation networks on multi-modal BraTS data included the original data and the augmented data, as shown in Table 3:

Table 3 shows that the dice score of all the three models with data augmentation outperformed the cases without augmentation. The average dice scores were improved by 2–3% for each of the segmentation models. All three models had a large improvement in tumor core segmentation. The performance of the whole-tumor and enhanced region segmentation tasks were also improved.

#### 4.4.2. Training on Single Modality Data of U-Net

Table 4 shows the efficiency of TumorGAN-based augmentation in single modality data-based segmentation tasks. Without the support from other modalities, the scores provided in Table 4 were lower than that reported in Table 3. Based on the results of Table 3 and Table 4, we can conclude that the TumorGAN-based augmentation method can enhance the dice scores for most segmentation tasks, in the case of single modality inputs.

**Figure 10:**
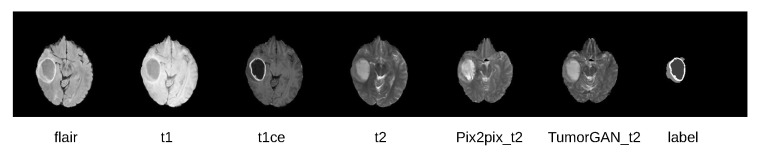
From left to right is the flair, t1, t1ce and t2 modality brain image from the real data. The pix2pix_t2 is the t2 modality image synthesized by the Pix2pix according to the same tumor. The TumorGAN_t2 is generated by TumorGAN. The last column is the segmentation label.

We evaluated the performance of various data augmentation methods on the brain tumor segmentation tasks with U-Net (See Table 4). The comparison analysis was done between our proposed TumorGAN and Pix2pix on four modalities (flair, t2, t1, and t1ce). In practical application, synthetic images are used to improve the segmentation accuracy. Obviously, most of the images generated by CycleGAN shown in Figure 8 do not have a clear tumor area. Thus, it would be meaningless to consider images generated by CycleGAN for comparison. It can be observed that augmenting dataset with both TumorGAN and Pix2pix generated images can enhance the segmentation performance in modalities including flair, t1 and t1ce. The performance obtained with TumorGAN was higher than that of Pix2pix, indicating that the images generated with the proposed TumorGAN can aid the segmentation task by providing more realistic tumor images as compared to Pix2pix. We also found that both TumorGAN and Pix2pix cannot improve the segmentation performance in t2. To investigate the cause regarding the performance degradation on t2, generated t2 images from both TumorGAN and Pix2pix and their ground truth segmentation label were shown in Figure 10. It can be observed that tumor regions including the “whole”, “core” and “en” can be clearly seen in flair, t1 and t1ce, and the boundary between “core” and “en” is also clear in these three modalities. However, the region of “en” can not be clearly visualized on the t2 images. Similarly, the synthesis t2 images from Pix2pix and TumorGAN also had a vague boundary between the “core” and “en”. Hence, the blurred boundary found on t2 images may cause performance to degrade when we use an image-to-image based augmentation method, as reflected on the poor segmentation performance on “en”.

## 5. Conclusions and Future Work

In this paper, we proposed a novel GAN-based image-to-image framework called TumorGAN for brain tumor image augmentation. By combining the brain tissue area and the tumor area from two different patients, the proposed method can create $n^{2}-n$ virtual data pairs from the data of *n* patients. To further improve the generation performance, we introduced a regional perceptual loss and a regional $L_{1}$ loss with a local discriminator. Compared with other GAN-based image-to-image translation frameworks, our method can create high-quality image pairs from limited paired data. As proved by the experimental results, the synthesis image pairs from TumorGAN can practically help to improve tumor segmentation in both multi-modal and single-modality data sets.

In our work, we note that reasonable virtual semantic labels are the key to generating realistic synthesized samples and, so, we tried to obtain a reasonable tumor region from the existing samples, providing more possible combinations between tumors and healthy tissue. However, our method cannot provide an unseen tumor label, which restricts the diversity of the virtual semantic labels. Fortunately, multiple GAN-based studies have indicated that the generated shape or style can be controlled by latent codes [13,50], which may help us to control the generated tumor shape and increase the diversity of the virtual semantic labels. We leave this for a future work.

## Figures and Tables

**Table 1:** Data split.

Data Sets	All	HGG	LGG
Total	285	210	75
Train	226	166	60
Augmentation	226	166	60
Test	59	44	15

**Table 2:** FID (lower is better). TumorGAN generates diverse and real images that compare favorably to those of the state-of-the-art baselines.

	CycleGAN (Baseline)	Pix2Pix	w/o per	w/o d_lcoal	TumorGAN
FID	154.86 (0%)	126.42 (18.36%)	87.75 (43.34%)	145.67 (5.93%)	77.43 (50%)

**Table 3:** Dice score comparison of different segmentation networks trained on “Train” and “Train + TumorGAN Augmentation”. All networks were trained without usual data augmentation techniques (e.g., flip, crop, rotation, and so on). The best mean dice scores are highlighted bold.

Networks		Whole	Core	en	Mean
Cascaded Net	Without augmentation	0.848	0.748	0.643	0.746
	With TumorGAN augmentation (ours)	0.853	0.791	0.692	**0.778**
U-Net	Without augmentation	0.783	0.672	0.609	0.687
	With TumorGAN augmentation (ours)	0.806	0.704	0.611	**0.706**
Deeplab-v3	Without augmentation	0.820	0.700	0.571	0.697
	With TumorGAN augmentation (ours)	0.831	0.762	0.584	**0.725**

**Table 4:** Dice score comparison of U-Net trained on single modality “Train”, “Train+Augmentation” and “Train + Pix2pix augmentation”. The best mean dice scores are highlighted bold

Modality		Whole	Core	en	Mean
flair	without augmentation	0.754	0.513	0.286	0.518
	with pix2pix augmentation	0.745	0.527	0.214	0.495
	with TumorGAN augmentation	0.765	0.522	0.289	**0.525**
t2	without augmentation	0.743	0.577	0.335	**0.552**
	with pix2pix augmentation	0.729	0.593	0.220	0.514
	with TumorGAN augmentation	0.750	0.572	0.321	0.548
t1	without augmentation	0.628	0.422	0.199	0.416
	with pix2pix augmentation	0.635	0.489	0.106	0.410
	with TumorGAN augmentation	0.628	0.467	0.235	**0.443**
t1ce	without augmentation	0.597	0.534	0.570	0.567
	with pix2pix augmentation	0.659	0.673	0.545	0.626
	with TumorGAN augmentation	0.671	0.681	0.589	**0.647**
